# Asymmetric Lipid Membranes under Shear Flows: A Dissipative Particle Dynamics Study

**DOI:** 10.3390/membranes11090655

**Published:** 2021-08-26

**Authors:** Yanying Chen, Zhenguo Wang, Yongyun Ji, Linli He, Xianghong Wang, Shiben Li

**Affiliations:** Department of Physics, Wenzhou University, Wenzhou 325035, China; cyyleslie@gmail.com (Y.C.); wzg@wzu.edu.cn (Z.W.); yyji@wzu.edu.cn (Y.J.); linlihe@wzu.edu.cn (L.H.); wangxh@wzu.edu.cn (X.W.)

**Keywords:** asymmetric, membrane, shear flow, dissipative particle dynamics

## Abstract

We investigate the phase behavior of the asymmetric lipid membranes under shear flows, using the dissipative particle dynamics simulation. Two cases, the weak and strong shear flows, are considered for the asymmetric lipid microstructures. Three typical asymmetric structures, the membranes, tubes, and vesicle, are included in the phase diagrams, where the effect of two different types of lipid chain length on the formation of asymmetric membranes is evaluated. The dynamic processes are demonstrated for the asymmetric membranes by calculating the average radius of gyration and shape factor. The result indicates that different shear flows will affect the shape of the second type of lipid molecules; the shape of the first type of lipid molecules is more stable than that of the second type of lipid molecules. The mechanical properties are investigated for the asymmetric membranes by analyzing the interface tension. The results reveal an absolute pressure at the junctions of different types of particles under the weak shear flow; the other positions are almost in a state of no pressure; there is almost no pressure inside the asymmetric lipid membrane structure under the strong shear flow. The findings will help us to understand the potential applications of asymmetric lipid microstructures in the biological and medical fields.

## 1. Introduction

A lipid molecule usually consists of one head chain and one or two more tail chains, which is an indispensable part of the human body and a vital component of various products, such as food, cosmetics, and medicines [1,2,3]. Due to the amphipathicity of head and tail chains in the aqueous solutions, lipid molecules can self-assemble into many different structures, such as the membrane, tube, vesicle, and a series of continuous structures. Among these lipid structures, the membrane, i.e., the lamellar structure, as the boundary between two diverse environments, is the main structure of the cell membranes, which arises wide concern [4]. In general, the lipid membranes are composed of various types of lipids, which extensively exist in the liquid environment [5]. Daniel et al. have researched a molecular organization and complex formation of FABT in DPPC multibilayers [6]. On the basis of the interpretation of Fourier transform infrared spectra, Dariusz Kluczyk et al. have analyzed the molecular organization of two compounds, that is, C1 and C7, in multilayers formed from DPPC and the 1,3,4-thiadiazoles [7]. By the vesicle fusion technique on mica, Seeger et al. have assembled the supported lipid bilayers composed of POPE and POPG [8]. Thus, the asymmetry and shear flows should be considered for the lipid membranes, which is beneficial to explore the mechanism of self-assembly and potential biological applications of asymmetric lipid microstructures.

Various symmetric lipid structures have been extensively investigated in previous works [9,10,11,12,13]. In order to obtain the special properties of the membrane, some studies have optimized and designed some new symmetric membranes. For example, Cheng et al. have designed symmetric, support-free, and self-standing osmotic membranes with exceptionally high mechanical robustness and desalination performance [12]. The lipid structure containing one hydrophilic functional group and one or two hydrophobic fatty acid groups is amphiphilic in aqueous environments. The single tail of fatty acid makes the molecule wedge-shaped, which is beneficial to the formation of micelles. The multiple tails of phospholipids make the molecules more cylindrical, allowing them to form planar bilayer sheets. Depending on their shape and the amphiphilic properties of lipids, lipid molecules form various microstructures spontaneously in aqueous solutions [9,10,11,13]. These experiments and simulations suggested that the bilayer lipids will spontaneously rearrange to eliminate the free edge because of the unfavorable energy; only by closing in on itself and forming a sealed compartment, the edges for the bilayer can be avoided.

Asymmetric lipid structures exist in nature and play a crucial role in living organisms. Because of the particularity of the asymmetric structures, they are often applied in many applications, such as interfacial mass transfer, switchable ion transport, and unidirectional oil/water separation [14,15,16,17]. Kakuda et al. have described protocols for preparing asymmetric lipid vesicles and have explained how they are applied to the behavior of pore-forming toxin [18]. Employing a pulsed-jet method, Kamiya et al. have produced nano-sized asymmetric lipid vesicles, which are produced by asymmetric planar lipid bilayers by applying longer duration and higher pressure pulsed-jet flows, rather than those used to form micro-sized lipid vesicles [19]. At present, many studies on asymmetric lipid bilayers have also been published [20,21,22,23,24,25,26,27]. For example, by the vesicle fusion technique on mica, Seeger et al. have assembled the supported lipid bilayers and the influence of different physical parameters on the main phase transition have also been clarified [8]. When introducing the shear flows, the lamellar microstructures can orient along the flow direction and enlarge the phase spaces for lipid membranes in the phase diagrams. These phase diagrams provide information on membrane structure distributions in the parameter spaces under various conditions at equilibrium states [28] (see 1. The equilibrium state in Supplementary Material File for more details). However, more studies are based on asymmetric lipid structures in the zero flow [29,30,31,32,33,34,35,36,37,38]. For example, there are novel functional paper membranes with adjustable gradients and Janus-type wettability. This asymmetric wettability is originated from an asymmetric silica distribution along the paper cross [30]. Furthermore, the recent experiments indicate that the biological cells react to the flow field around them, changing their activities in the physiological environment [39]. We note that the previous studies mainly concentrated on either the symmetric lipid structures in zero flows or the asymmetric lipid structures in the shear flows. It is still worthwhile to further explore the formation mechanism for the asymmetric lipid structures formed by different lipids in shear flows, which may have potential applications in biological areas.

In the current study, we analyze the asymmetric microstructures composed of two types of lipids and investigate the dynamic processes of lipid structures in aqueous solutions using the dissipative particle dynamics (DPD) simulation based on the coarse-grained (CG) model. We calculate phase diagrams in terms of different chain lengths under three different flows condition observing the formation of asymmetric membranes, tubes, and vesicles. Moreover, we are interested in the dynamic processes and mechanical properties of asymmetric lipid membrane structures. Section 2 presents the method and model, Section 3 emerges the results and discussions and Section 4 shows the summary.

## 2. Method and Model

### 2.1. Method

By taking a group of atoms as one particle(bead), also known as the CG model, the DPD method simulates the hydrodynamic behavior of the complex fluids [38,40,41,42,43,44]. Based on following Newton’s law, three types of forces, the conservation force, dissipative force, and random force, are introduced to describe the movement of particles between the *i*-th and *j*-th pairs. Conservation force $F_{ij}^{C}$ is introduced to eliminate the volume effect. Dissipative force $F_{ij}^{D}$ represents the viscous resistance among moving particles in order to decrease the radial velocity difference. Random force $F_{ij}^{R}$ typifies a stochastic force. Based on Newton’s laws of motion, all these forces between the *i*-th and *j*-th pairs along the line between the centers of these two particles, each of which is pairwise additive:

(1) $$\frac{dr_{i}}{dt}=v_{i}$$

(2) $$\frac{dv_{i}}{dt}=f_{i}$$

(3) $$F_{i}=\sum_{i\neq j}\left(F_{ij}^{C}+F_{ij}^{D}+F_{ij}^{R}\right)$$

where

$$\begin{array}{c} F_{ij}^{C}=a_{ij}w\left(r_{ij}\right){\hat{r}}_{ij}\\ F_{ij}^{D}=-\gamma w^{2}\left(r_{ij}\right)\left({\hat{r}}_{ij}\cdot v_{ij}\right){\hat{r}}_{ij}\\ F_{ij}^{R}=\sigma w\left(r_{ij}\right)\zeta_{ij}\Delta t^{-0.5}{\hat{r}}_{ij} \end{array}$$

$a_{ij}$ signifies the maximum repulsive force, which is related to the Flory-Huggins χ-parameter. $r_{ij}$ = $r_{i}-r_{j}$ and $v_{ij}$ = $v_{i}-v_{j}$ represent the relative position and velocity, respectively. ${\hat{r}}_{ij}$ = $r_{ij}/\left|r_{ij}\right|$ is the unit vector, γ is the friction coefficient, and σ is the noise amplitude. γ and σ are related as $\sigma^{2}$ = $2\gamma k_{B}T$, where *T* is the absolute temperature, and $k_{B}$ is the Boltzmann constant. $\zeta_{ij}$ denotes a random number from a uniform random distribution with unit variance and Gaussian distribution. σ=3.0 and γ=4.5 are usually used as standard values in the simulation. In order to attain a more accurate speed calculation, this simulation uses the Velocity–Verlet algorithm [45]:

(4) $$\begin{array}{c} r_{i}\left(t+\Delta t\right)=r_{i}\left(t\right)+\Delta tv_{i}\left(t\right)+\frac{1}{2}{\left(\Delta t\right)}^{2}f_{i}\left(t\right)\\ {\tilde{v}}_{i}\left(t+\Delta t\right)=v_{i}\left(t\right)+\lambda \Delta tf_{i}\left(t\right)\\ f_{i}\left(t+\Delta t\right)=f_{i}\left(r\left(t+\Delta t\right),{\tilde{v}}_{i}\left(t+\Delta t\right)\right)\\ v_{i}\left(t+\Delta t\right)=v_{i}\left(t\right)+\frac{1}{2}\Delta t\left(f_{i}\left(t\right)+f_{i}\left(t+\Delta t\right)\right) \end{array}$$

The weight function $w\left(r_{ij}\right)$ is expressed by the following formula:

(5) $$w\left(r_{ij}\right)=\left\{\begin{array}{cc} 1-\frac{r_{ij}}{r_{c}} & ,r_{ij}<r_{c}\\ 0 & ,r_{ij}>r_{c} \end{array}\right.$$

where $r_{c}$ is the cut-off radius.

### 2.2. Model

**Figure 1:**
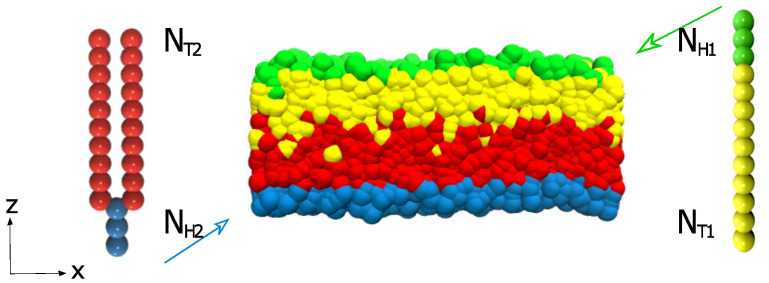
Two types of lipid molecules and the asymmetric structure are displayed in the schematic diagram. An example of the asymmetric membrane formed by these two types of lipids is exhibited in the center.

There are many asymmetric structures worth studying in the simulation, which are generally divided into the same type of structures and different types of structures. This paper focuses on the asymmetric bilayer structures formed by lipid structures of different configurations. Because of the complexity of the molecule, the simple CG model in DPD is used. As shown in Figure 1, there are two different lipids forming the asymmetric lipid structure. The first type of lipids with one head and one tail is demonstrated on the right, where the greens and yellows denote the head and tail beads, respectively. The second type of lipids with one head and two tails is shown on the left, where the blues and reds represent the head and tail particles, respectively. These consecutive particles are in connection with an additional elastic harmonic force:

(6) $$F_{ij}=k_{s}\left(1-\frac{r_{ij}}{r_{s}}\right){\hat{r}}_{ij}$$

where $k_{s}$ and $r_{s}$ are the spring constant and equilibrium bond length, singly. Based on previous works, we adopt $k_{s}=120.0$ and $r_{s}=0.7r_{c}$ in the current simulation. An extra force caused by a harmonic constraint on two consecutive shows the bending force of the lipid molecule:

(7) $$F^{\theta }=-\nabla \left[k_{\theta }{\left(\theta -\theta_{0}\right)}^{2}\right]$$

where $k_{\theta }$, $\theta_{0}$ and θ are the bending constant, the equilibrium angle, and the inclination angle, respectively. We use $k_{\theta }=6.0$ and $\theta_{0}=\pi$ for three consecutive head particles and three consecutive tail particles in each chain for the first type of lipids with one head and one tail. As for the second type of lipids with one head and two tails, we use $k_{\theta }=6.0$ and $\theta_{0}=\pi$ for three consecutive head particles and three consecutive tail particles in each chain; $k_{\theta }=3.0$ and $\theta_{0}=\frac{2}{3}\pi$ for the last two consecutive head particles and the first tail particle; $k_{\theta }=4.5$ and $\theta_{0}=\frac{2}{3}\pi$ for the last head particle and the first two tail particles. We adopt the above data based on many previous studies [40,46,47]. However, the asymmetric structure of lipid molecules has been studied more before; we select two different types of lipids, which are precisely different from the previous works.

### 2.3. Shear Flow

**Figure 2:**
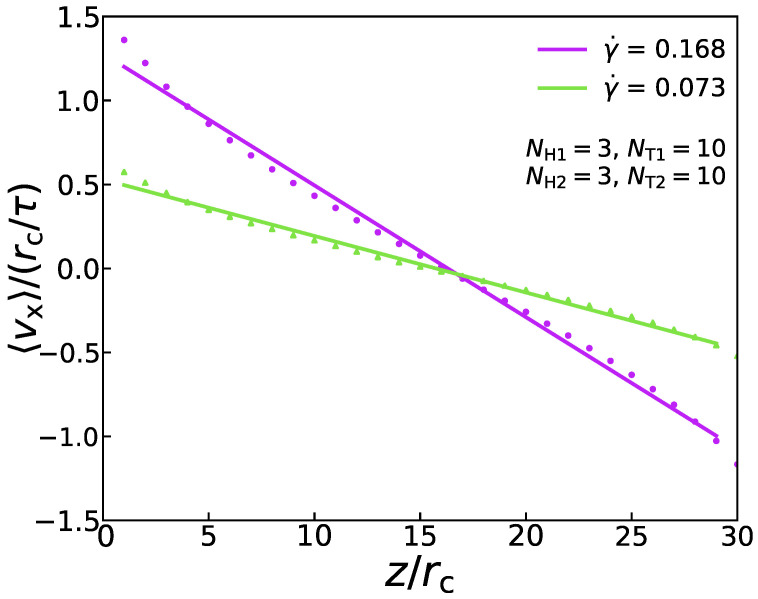
An example of velocity profile at $\dot{\gamma }$ = 0.168 and $\dot{\gamma }$ = 0.073 in the simulation box.

In the DPD simulation, a nonequilibrium method is used to calculate the shear viscosity that connects a shear field with a flux of transverse linear momentum [48,49,50]. In the current simulation, the shear field is a gradient in $v_{x}$ set up in the direction by shearing the liquid, denoted as the shear rate: $\partial v_{x}/\partial z$. The momentum flux $j_{z}\left(p_{x}\right)$ is generated by *x* momentum flows in the direction per given time and per unit area, which is followed as:

(8) $$j_{z}\left(p_{x}\right)=-\eta \frac{\partial \nu_{x}}{\partial z}$$

where η signifies the shear viscosity. In the DPD simulation, this momentum flux is applied to the system in an unphysical way [49]. To be specific, the periodic analog box with side length $L_{z}$ is divided into several slabs along the z coordinate. The atoms inside the slabs, at z=0 and $z=L_{z}$, are pushed in two opposite x directions, respectively. By finding the atom most moving against the desired slab movement, the atom with the largest and smallest $p_{x}$ in the *x*-direction can be found. As a result of two atoms with the same mass and their location remains the same, the potential energy and the system’s total energy are conserved. Hence, the $p_{x}$ exchange between the two particles is achieved. As an example, Figure 2 shows that the velocity distribution $\langle v_{x}\rangle$ conforms to a specific linear relationship under the two shear rates, which proves the rationality of this method. Because of the momentum exchange’s periodicity, the sum of the $\Delta p_{x}$ equals the total momentum transferred in a simulation. In addition, the momentum exchange rate by momentum swaps is equal to that of momentum flowing in the opposite direction via a physical mechanism (friction) [49]. Therefore, the Equation (8) can also be expressed as:

(9) $$j_{z}\left(p_{x}\right)=\frac{p_{x}}{2tL_{x}L_{y}}$$

where *t* is the length of the two swaps. $L_{x}$ and $L_{y}$ represent the length of two sides of the periodic box in the x- and y-directions, respectively. Different shear flows can be obtained by adjusting the time of swaps to control the magnitude of momentum flux. In the current simulation, *W* is set as 6 and 1 to correspond to the weak shear flow ($\dot{\gamma }=0.073$) and the strong shear flow ($\dot{\gamma }=0.168$), respectively (see 2. The shear flow in Supplementary Material File for more details).

### 2.4. Parameters

In our simulations, we fix some parameters such as the mass of molecules, the cut-off radius, the time step, and the repulsive interaction parameters. For the repulsive interaction parameters, we set $a_{ii}=25$ and $a_{ij}=100$ for the same and different types of particles in the simulations, which is applied extensively in the previous works [45,51,52]. The formula of the cut-off radius is $r_{c}$ = ${\left(\rho V_{p}\right)}^{1/3}$, where ρ and $V_{p}$ typify the particle density and the volume of individual particle. According to the previous work, the volume of DPD particle is assumed as $V_{p}=0.03$ nm${}^{3}$; with reference to the previous researches, we set the parameter ρ=3 [45,53], i.e., we assume $r_{c}$ approximately to be 0.5 nm. In the simulations, the length is scaled as the normalized unit $r_{c}$, while the time is scaled as the normalized unit τ, which is defined as follows [54]:

(10) $$\tau =r_{c}\sqrt{m/k_{B}T}$$

Here, *m* is the bead mass, and $k_{B}T$, with Boltzmann constant $k_{B}$ and temperature *T*, is the energy scale. The timestep Δt=0.01τ is taken as following a modified velocity Verlet algorithm, where τ=1.88 ns, i.e., Δt=0.0188 ns [45].

Using large-scale atomic/molecular massively parallel simulator (LAMMPS), our simulations are carried out on the NVT ensemble systems by fixing the number of particles, volume, and temperature of the system. By setting the periodic boundary conditions of three directions, the simulation is limited to a cube with a side length *L*. In the simulations, the size of the simulated box is optimized as $L=30r_{c}$ by changing from $25r_{c}$ to $35r_{c}$ to avoid the finite-size effect [29,55]. We fix the numbers of chains for both lipids as $n_{1}=n_{2}=600$, and the number of head particles for two lipids is 3. We set total DPD time steps as 200,000 in the dynamic process to obtain equilibrium structures similar to that in the former study [29]. We set up multiple initial states to acquire a structure with the minimum energy value. Then, we select this structure as the most stable structure in the simulations.

## 3. Results and Discussion

**Figure 3:**
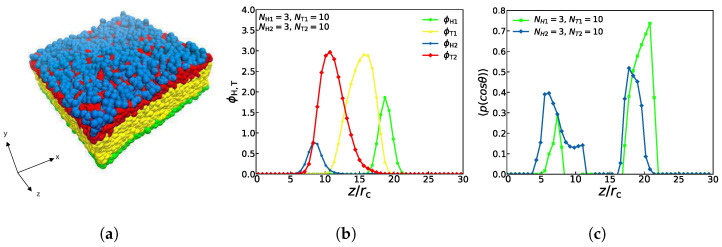
A snapshot for the lipid bilayer (**a**) is shown at the top. The density profiles (**b**) of head and tail particles and the order parameters (**c**) are shown along the z axes in the middle and bottom, respectively.

**Figure 4:**
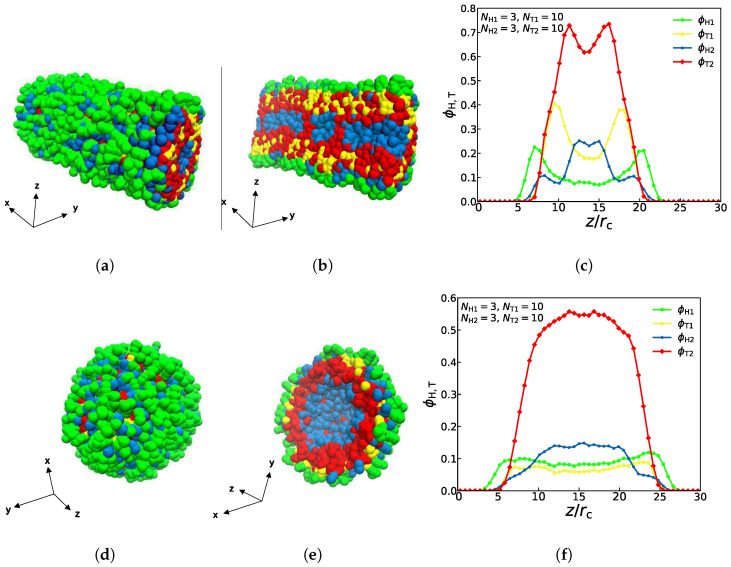
Typical asymmetric tube and vesicle. (**a**–**c**) asymmetric tube with $N_{H}1=3$, $N_{T}1=5$, $N_{H}2=3$, $N_{T}2=5$, (**d**–**f**) asymmetric vesicle with $N_{H}1=3$, $N_{T}1=2$, $N_{H}2=3$, $N_{T}2=6$. The density profiles of head and tail particles are shown along the *z* axes on the right.

**Figure 5:**
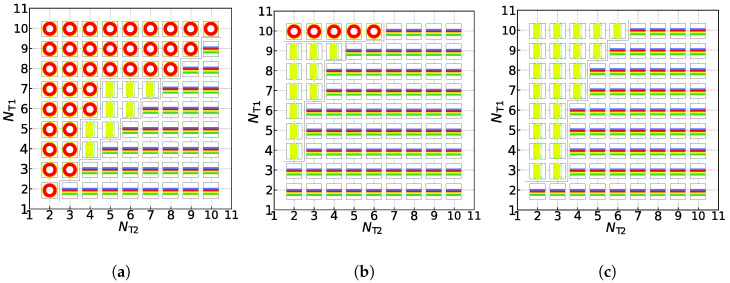
The phase diagrams in (**a**) the zero shear flow($\dot{\gamma }$ = 0), (**b**) the weak shear flow ($\dot{\gamma }$ = 0.073), and (**c**) the strong shear flow ($\dot{\gamma }$ = 0.168). The phase symbols 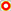 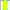 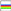 represent the asymmetric vesicle, asymmetric tube and asymmetric membrane, respectively.

**Figure 6:**
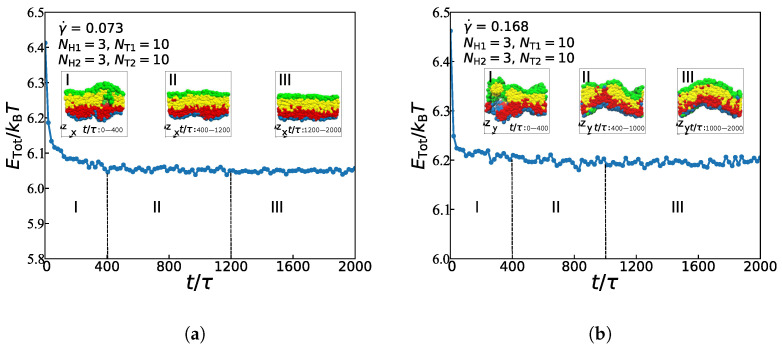
The total energy as functions of time steps for the asymmetric membranes under (**a**) $\dot{\gamma }$ = 0.073 and (**b**) $\dot{\gamma }$ = 0.168.

**Figure 7:**
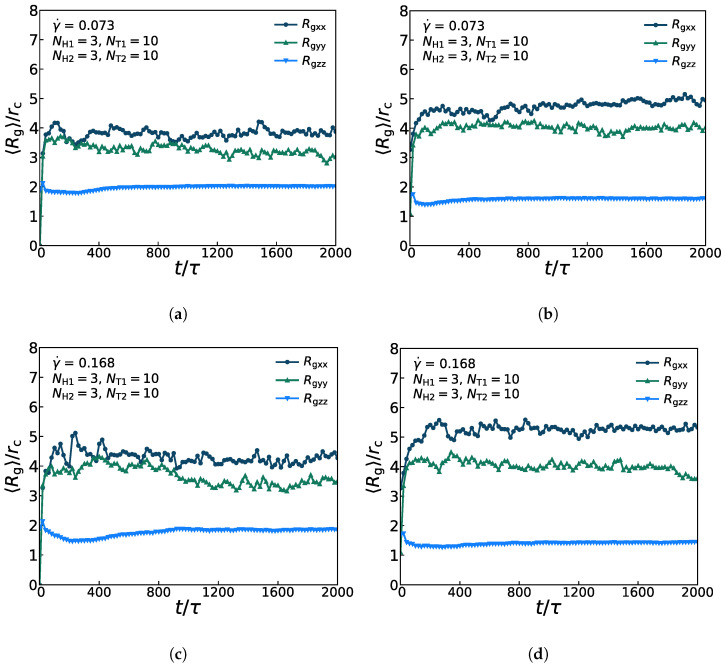
The average radius of gyration of the asymmetric membrane. There are three components $R_{\mathrm{gxx}}$, $R_{\mathrm{gyy}}$, $R_{\mathrm{gzz}}$ of $R_{g}$ at two different shear rates $\dot{\gamma }$ = 0.073 and $\dot{\gamma }$ = 0.168 as functions of time steps for (**a**,**c**) the first type of lipid chains, and (**b**,**d**) the second type of lipid chains.

**Figure 8:**
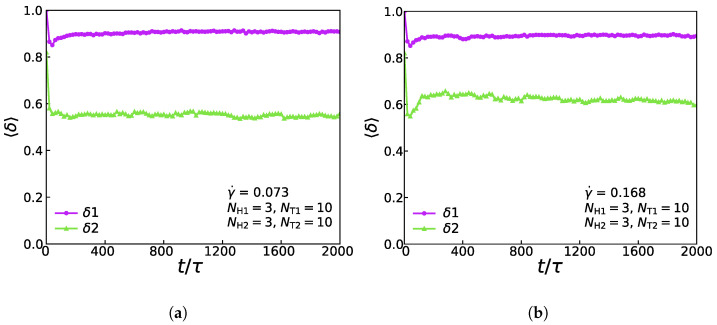
The shape factor of the asymmetric membrane for the first type of lipid chain (δ1) and the second type of lipid chain (δ2) with (**a**) $\dot{\gamma }$ = 0.073 and (**b**) $\dot{\gamma }$ = 0.168.

**Figure 9:**
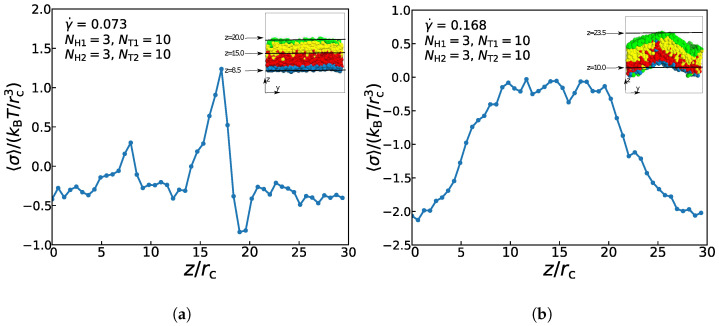
The interface tension 〈σ〉 as functions of distance along *z*-direction for the asymmetric membrane in the case of (**a**) $\dot{\gamma }$ = 0.073 and (**b**) $\dot{\gamma }$ = 0.168. The snapshots of asymmetric membranes are also inserted.

Following the above model and method, the phase space includes a series of parameters, such as the repulsive interaction parameters ($a_{ij}$), the numbers of molecular chains of lipids ($n_{1},n_{2}$), the bond length ($0.5r_{c}$), and the numbers of head and tail particles of lipids ($N_{H}1,N_{H}2,N_{T}1,N_{T}2$). It is difficult to observe all the variables simultaneously, so we pay more attention to distinguishing the dynamic processes and mechanical properties caused by the change of the number of tail particles of lipids, $N_{T}1,N_{T}2$. Namely, we vary $N_{T}1$ and $N_{T}2$ for two types of lipids from 2 to 10 under the weak and strong shear flows while fixing the other parameters as mentioned in the above section. Figure 3 and Figure 4 display three main asymmetric structures produced from our simulations; phase diagrams are shown in Figure 5; the dynamic processes are demonstrated in Figure 6, Figure 7 and Figure 8; the mechanical properties are exhibited in Figure 9.

### 3.1. Asymmetric Structures

This subsection mainly introduces three categories of asymmetric structures: the asymmetric membrane (i.e., asymmetric lamellar structure), the asymmetric tube (i.e., asymmetric columnar structure), and the asymmetric vesicle (i.e., asymmetric spherical structure). These structures formed by the self-assembly of two types of lipid molecules introduced previously in aqueous solutions have good structural stability [29].

An asymmetric membrane, with $N_{H}1=3$, $N_{T}1=10$, $N_{H}2=3$, and $N_{T}2=10$ is shown in Figure 3. Assuming that this structure is filled with aqueous solutions, it follows that the hydrophilic head particles (the blue and green beads) are distributed in the outer side of the structure while the hydrophobic tail particles (the red and yellow beads) are distributed in the inner side of the structure, which is displayed in Figure 3a. Obviously, these distributions are due to the attractive interactions between the head particles and water particles, and the repulsive interactions between the tail particles and the water particles. Figure 3b demonstrates the particle density profiles of the hydrophilic and hydrophobic particles along the *z*-direction (see 3. The method of density calculation in Supplementary Material File for more details). There are four peaks in the density distribution, and the curves of the different colors correspond to the four types of particles in Figure 3a. The blue curve peak of the head particle representing the second type of lipid molecules and the green curve peak of the head particle representing the first type of lipid molecules appear first and last, which indicates that the hydrophilic particles of the head are located in the uppermost and lowest layers of the structure, respectively. The peaks of these two tail particles(the red and yellow curves) appear one after another and overlap partially, demonstrating that the tail chains are interdigitated partially for these two types of lipids. This density distribution diagram exhibits the structure of the asymmetric membrane, which has been confirmed in the previously reported lipid membrane structures [56,57].

In addition, an order parameter can be used to demonstrate the orientational ordering of the head particles for this asymmetric membrane [58,59]:

(11) $$\langle P\left(\cos\theta \right)\rangle =\langle \frac{3}{2}\cos^{2}\theta -\frac{1}{2}\rangle$$

where θ is the angle between the chain and *z* coordinate direction, and the single quotation marks express an ensemble average. Since the rigidity of the head particles in the lipid structure in this paper is much greater than that of the tail particles, we only calculate the order parameters of the head particles. When the orientation ordering value is equal to 1, the chain direction is entirely parallel to the *z* coordinate direction; when the orientation ordering value is equal to 0.50, the chain direction is utterly perpendicular to the *z* coordinate direction. The value of 0.0 represents random orientation [40]. Figure 3c shows the orientation distributions, where the green and blue dots signify the simulation results of the first and second type of head particles of lipids. With an ordering value of about 0.40, the second type of lipid molecules is mainly distributed between $3r_{c}$ and $12r_{c}$, while with an ordering value of about 0.74, the first type of lipid molecules are mainly distributed between $17r_{c}$ and $22r_{c}$, which means that the angles between these two types head chains and the *z* coordinate direction range from 0 to π. This phenomenon indicates that asymmetric membranes have well-ordered structures in the interfacial regions. The order peak value of the first type is close to 1, indicating that the head chain of the first type of lipids is almost parallel to the *z* axis. The first type of lipids has far more chains than the second type, which verifies that the number of chains also affects the value of the ordering parameter so that the lipid molecules with more chains have the higher ordering in the lipid-water mixtures [28].

The density profiles of the other two asymmetric structures (asymmetric tube and asymmetric vesicle) are also observed and studied, as shown in Figure 4, where both the complete schematic diagrams and the sectional drawings are displayed. With parameters of $N_{H}1=3$, $N_{T}1=5$, $N_{H}2=3$ and $N_{T}2=5$, we can observe that the columnar structure has the characteristics of asymmetry, the particles of the first type of lipids are mostly distributed in the outer layer while particles of the second type of lipids are mostly distributed in the inner layer, as shown in Figure 4a,b. This distribution is mainly caused by the amphipathicity of lipid chains in the aqueous solutions. Notably, four different colors of curves in Figure 4c represent four different types of particles, and the yellow and green particles representing the first type of lipids both reach a low value in the interior of the structure, namely, the interior of the columnar structure contains very few of the first type of lipids, which is consistent with former works [29]. Similarly, with parameters of $N_{H}1=3$, $N_{T}1=2$, $N_{H}2=3$, and $N_{T}2=6$, the spherical structure also has the characteristics of asymmetry, which is shown in Figure 4d,e. Particles of the first type are mostly distributed in the outer layer, while particles of the second type are mostly distributed in the inner layer. Obviously, four different colors of curves in Figure 4f represent four different types of particles, and the red and blue particles representing the second type of lipids both reach a high value in the middle, especially the number of hydrophobic red particles was significantly higher than the number of particles of any other color, which means that the spherical structure is consistent with the characteristics of hydrophilic head particles and hydrophobic tail particles of lipids. Certainly, the amphiphilic chains lead to the distribution sequence of the tail and head parts in the aqueous solution. The previous works also reported the self-assembly structures of asymmetric vesicle [29,33]. Here, we observed the asymmetric vesicle constructed by two different types of lipid molecules in the aqueous solutions due to the amphiphilic interactions.

### 3.2. Phase Diagrams

Now, we turn to the effect of chain length on the formation of the asymmetric membrane. We construct the phase diagrams with $N_{H}1=3$ and $N_{H}2=3$ for three candidate structures, the asymmetric membrane, tube, and vesicle, based on the tail chain length for the two types of lipids, i.e., $N_{T}1$ and $N_{T}2$. Figure 5 exhibits the phase diagrams constructed following the minimum free-energy selection: the structural distributions of three asymmetric structures in the conditions of the zero shear flow, weak shear flow, and strong shear flow. Under the zero shear flow, as shown in Figure 5a, there is a clear diagonal distinction between these three structures. Asymmetric membranes are all distributed below the diagonal line, while the asymmetric tubes and vesicles are all distributed above the diagonal line. In other words, with the increase in tail particles of the second type of lipids, asymmetric membranes are more easily formed under the zero shear flow. Conversely, asymmetric vesicles are more simply formed with the decrease in tail particles of the second type of lipids. When the number of tail particles of the two lipids is close to each other, asymmetric tubes are more stable, which embodies that asymmetric tubes are distributed in the middle of this phase diagram. Under the same parameter conditions, we apply a weak shear flow with $\dot{\gamma }=0.073$ to these three structures, which is shown in Figure 5b. Contrasting that with the phase diagram at the zero shear flow, it can be observed that, except for the partial asymmetric vesicles of $N_{T}1=10$, other asymmetric vesicles are shear-induced into columnar structures when the number of particles in a tail of the second type of lipids is small, and shear-induced into asymmetric membranes when the number of particles in a tail of the second type of lipids is large. This is because the first type of lipid molecules’ tail particle number, also known as the number of outer particles of the spherical structure, is not enough, and asymmetric vesicles themselves are relatively less stable, which leads to their deformations under the weak shear flow. However, under the strong shear flow, we can observe that there are only two kinds of structures, the asymmetric membranes, and the asymmetric tubes, in the phase diagram, as shown in Figure 5c. Asymmetric vesicles and some asymmetric membranes gradually transform into asymmetric tubes by the strong shear force ($\dot{\gamma }=0.168$). As the number of tail particles of the second type of lipid molecules increases, asymmetric membranes can still maintain their structures under the strong shear flow, which confirms that these structures are always stable. Here, the phase diagrams provide the hybrid effects of tail chain length and shear flow strength on the asymmetric membranes, which differ from the previous simulations where only consider either chain length or shear flow strength [28,29,40].

### 3.3. Dynamic Processes

In this subsection, we focus on the dynamic processes to understand the formation mechanism of the asymmetric lipid membranes in the weak and strong shear flows by fixing the chain length with $N_{H}1=3$, $N_{T}1=10$, $N_{H}2=3$, and $N_{T}2=10$. Figure 6 illustrates the energy changes with a time step and the main evolutionary states under the weak and strong shear flows, where $\dot{\gamma }$ = 0.073 and 0.168, respectively. In both cases, the process of structural evolution can be divided into three parts: the random generation stage, the mutual adaptation stage, and the formation stage [29]. In the weak flow case with $\dot{\gamma }$ = 0.073, as shown in Figure 6a, the asymmetric membrane is in the random stage between the time *t* of 0 and 400τ. In this random generation stage, free energy presents a trend of rapid decline. Additionally, the structure has changed a lot. Then, when *t* is from 400τ to 1200τ, the free energy shows a weak decline trend, where the asymmetric membrane is in the mutual adaptation stage. At this stage, the structure is still in a state of continuous adjustment and evolution. In the final formation stage, the asymmetric membrane’s free energy is maintained at a correspondingly stable value, which means that the structure already reaches an equilibrium state. Several previous studies have reported a similar evolutionary process in either the symmetric or asymmetric lipid membranes under the zero shear flow [29,40]. Here, we observed the dynamic process for the asymmetric membranes under the weak shear flows, indicating that the weak shear flows rarely affect the dynamic process for the structural formation.

However, when the shear rate is increased to be $\dot{\gamma }$ = 0.168, the strong shear flow, the dynamic process differs from that in the weak shear flow, as shown in Figure 6b. In the random stage between the time of 0 and 400τ, the structure’s shape change is pronounced, and the free energy shows a rapid decline trend. Later, the asymmetric membrane is in the mutual adaptation stage with a time *t* of 400τ to 1000τ. The structure is still in continuous adjustment and evolution in this phase, and its free energy shows a slow decline. The energy tends to stabilize during the final formation stage, which indicates that the asymmetric membrane has already reached a stable structure in this phase. By comparing the dynamic evolution processes of the asymmetric membrane under the strong and weak shear flows, it can be seen that the random generation stage of the asymmetric membranes are almost the same under these two conditions; while the mutual adaptation stage of the asymmetric membrane becomes shorter under the strong shear flow, that is to say, the asymmetric membrane becomes stable faster under the strong shear flow than it under the weak shear flow. By observing the final morphology under these two shear flows, it demonstrates that the asymmetric membrane is more smooth under the weak shear flow, while the asymmetric membranes will form a certain degree of bending along the shear direction under the strong shear flow. According to previous studies, in symmetric bilayers, the spontaneous curvature of the membrane is zero, because any inherent curvature of one leaflet is completely offset by an identical, but reversely oriented, opposing leaflet [60]. This spontaneous, or intrinsic, the curvature is due to the chemical structures of the constituent lipids. However, asymmetrically distributed lipids have very disparate spontaneous curvature, and there may be a non-zero energetic consumption to keep the bilayers flat, which is why the membrane with lower spontaneous curvature (Figure 6a) ends up with higher energy at a steady-state than the membrane with higher spontaneous curvature (Figure 6b). It also means that the smooth membrane structure is more stable than the crooked one, which is consistent with the previous simulations [29]. In addition, some studies have shown that lipid-lipid interaction is strongly coupled with curvature [61]; in other words, the surrounding environment of lipids will affect its curvature preference. Along the z-axis direction under the action of shear flow, the flat membrane structure will be a certain degree of bending along the shear direction; It is obvious that the greater the shear force, the greater the bending degree, which is consistent with the different characteristics of curvature of membranes in different shear flows environments in this paper. After a certain period, taking the membrane as the boundary, the shear rate on the membrane will have a certain difference from the shear rate under the membrane. The velocity difference under strong shear is large enough to affect the bending rate of the membrane structure to a certain extent. This result is of significance for the study of cellular function under different environments.

In order to describe the dynamic processes for the asymmetric membrane in more detail, we introduce the physical quantity of the average radius of gyration $\langle R_{g}\rangle$ [62], where the radius of gyration tensor $R_{g}^{2}$ can be expressed as:

(12) $$R_{g}^{2}=\left(\begin{array}{ccc} R_{gxx}^{2} & R_{gxy}^{2} & R_{gxz}^{2}\\ R_{gyx}^{2} & R_{gyy}^{2} & R_{gyz}^{2}\\ R_{gzx}^{2} & R_{gzy}^{2} & R_{gzz}^{2} \end{array}\right)$$

Thereinto, the element of $R_{g\alpha \beta }^{2}$ follows as:

(13) $$\langle R_{g\alpha \beta }^{2}\rangle =\frac{1}{N}\sum_{i}\langle \left(r_{i,\alpha }-r_{c,\alpha }\right)\left(r_{i,\beta }-r_{c,\beta }\right)\rangle$$

with α,β∈{x,y,z}, where $r_{i,\alpha }$ and $r_{c,\alpha }$ represent the α-coordinate of the *i*-th particle and the center of mass, respectively; and *N* is the number of chains (see 4. The gyration tensor in Supplementary Material File for more details).

Figure 7 presents the anisotropic conformations for lipid molecules in the diversified dynamic processes under the weak and strong shear flows, in which the asymmetric membrane is with parameters of $N_{H}1=3$, $N_{T}1=10$, $N_{H}2=3$, and $N_{T}2=10$. Figure 7a,c show the three components $R_{\mathrm{gxx}}$,$R_{\mathrm{gyy}}$,$R_{\mathrm{gzz}}$ of $\langle R_{g}\rangle$ for the first type of lipid chains with $\dot{\gamma }$ = 0.073 and $\dot{\gamma }$ = 0.168. The average values of $\langle R_{gxx}\rangle$ and $\langle R_{gyy}\rangle$ are relatively close and greater than those of $\langle R_{gzz}\rangle$, which indicates that the lipid molecules are relatively stretched in the *x*- and *y*-directions but compact in the *z*-direction. Figure 7b,d present the three components $R_{\mathrm{gxx}}$,$R_{\mathrm{gyy}}$,$R_{\mathrm{gzz}}$ of $\langle R_{g}\rangle$ for the second type of lipid chains with $\dot{\gamma }$ = 0.073 and $\dot{\gamma }$ = 0.168. Similarly, the average values of $\langle R_{gxx}\rangle$ and $\langle R_{gyy}\rangle$ are also relatively close and greater than those of $\langle R_{gzz}\rangle$. In addition, it also indicates that the lipid molecular model is stratified in the *x*-*y* plane under both the weak and strong shear flows. Then, as for the two types of lipid molecular models, the three components of $\langle R_{g}\rangle$ of the first type of the lipid model are much closer than the that of the second type of the lipid model, which substantiates that the amplitude of variation for first type of lipids is less than that for the second type of lipids (shown in Figure 6). By comparing the variation of $\langle R_{g}\rangle$ under the strong and weak shear flows, the oscillation amplitude of the curve, especially of the $R_{\mathrm{gxx}}$ and $R_{\mathrm{gyy}}$ under the strong shear flow is larger than that under the weak shear flow, which is in accordance with the comparison of structural changes under the two shear strengths. Our simulation results supported the argument that the strong shear flow can induce crystallization of lipids in the previous work [63].

In order to comprehend the changes of individual lipid molecules more clearly, we extract the shape factor expression of the structure through the radius of gyration tensor expression [64,65,66]:

(14) $$\langle \delta \rangle =1-3\langle \frac{L_{1}^{2}L_{2}^{2}+L_{2}^{2}L_{3}^{2}+L_{1}^{2}L_{3}^{2}}{{\left(L_{1}^{2}+L_{2}^{2}+L_{3}^{2}\right)}^{2}}\rangle$$

where $L_{1}^{2}$, $L_{2}^{2}$ and $L_{3}^{2}$ are the three eigenvalues of $R_{g}^{2}$. The shape factor 〈δ〉 ranges from 0 (the spherically symmetric objects) to 1 (the extremely elongated or ’cigar’ shaped objects) [64] (see 5. The shape factor in Supplementary Material File for more details).

Figure 8 shows the relationship of the shape factor (two types of lipids) for the asymmetric membrane with $N_{H}1=3$, $N_{T}1=10$, $N_{H}2=3$, and $N_{T}2=10$ in the cases of $\dot{\gamma }=0.073$ and $\dot{\gamma }=0.168$. In the weak shear flows with $\dot{\gamma }=0.073$, as shown in Figure 8a, the first type of lipid molecule is like a rod due to its initial configuration, which means that the initial value of the shape factor is 1.0. Soon there is a rapid drop and a slight rise back to a stable value of around 0.9, which exposes that when the simulation reaches equilibrium, the first type of lipid molecule’s shape is still like a rod. However, the shape factor of the second type of lipid molecule’s initial value is around 0.81, which is following its initial configuration. The shape factor increases first and then stabilizes at around 0.6, signifying that the chain exhibits an elliptical formation at the stable stage. Figure 8b shows the change of shape factor value with time step under the strong shear flow with $\dot{\gamma }=0.168$. By comparing the shape factors under the strong and weak shear flows, we can conclude that the shape factors of the first type of lipid molecules are approximately equal under two conditions of shear flows, while the value of the shape factors of the second type of lipid molecules under the strong shear flow is greater than that under the weak shear flow. This phenomenon indicates that different shear strengths will affect the shape of the second type of lipid molecules distinctly, while the shape of the first type of lipid molecules is more stable than that of the second type. We can notice that the result is similar to the previous simulation works [40].

### 3.4. Mechanical Properties

In this subsection, we concentrate on the mechanical properties of asymmetric membrane with $N_{H}1=3$, $N_{T}1=10$, $N_{H}2=3$, and $N_{T}2=10$ in the cases of $\dot{\gamma }=0.073$ and $\dot{\gamma }=0.168$. We quantitatively analyze the mechanical properties by calculating the interfacial tension. The Irving–Kirkwood theory defines the tension formula $\sigma_{z}$ along the *z* direction [67,68,69]:

(15) $$\sigma_{z}=\langle p_{zz}\rangle -\frac{1}{2}\left(\langle p_{xx}\rangle +\langle p_{yy}\rangle \right)$$

where the component of the pressure tensor $p_{xx}$ can be achieved as:

(16) $$p_{xx}=\frac{1}{V}\langle \sum_{i=1}^{N_{p}}m_{i}v_{ix}v_{ix}+\sum_{i=1}^{N_{p}}\sum_{j>i}^{N_{p}}F_{ijx}x_{ij}\rangle$$

where *V* is the volume of the box and $N_{p}$ is the number of the DPD particles. Here, $F_{ijx}$ and $x_{ij}$ represent the force and the relative position between *i*-th and *j*-th particles in the *x*-direction. The components $p_{yy}$ and $p_{zz}$ have the same definition as Equation (16) only by replacing the corresponding subscripts (see 6. Tension in Supplementary Material File for more details).

Figure 9 exemplifies the interface tension $\langle \sigma_{z}\rangle$ as functions of distance along *z*-direction for the asymmetric membrane with $N_{H}1=3$, $N_{T}1=10$, $N_{H}2=3$ and $N_{T}2=10$ in the two cases of $\dot{\gamma }$ = 0.073 and $\dot{\gamma }$ = 0.168. At *z* = 8.5$r_{c}$ and *z* = 20.0$r_{c}$, there is an appreciable change of the surface tension, while the change direction is opposite. These two points are the junctions of lipid molecules and the aqueous solutions, so there is an absolute pressure at the interface of the lipid membrane and the aqueous solutions towards the internal direction of the membrane. Between *z* = 12.0$r_{c}$ and 19.0$r_{c}$, the interfacial tension increases and decreases rapidly. In a state of equilibrium, the symmetrical bilayer structure is tensionless, while the internal stress of the asymmetric membrane is caused by the intrinsic stress of the bilayer structures, which is from the different packing of the two layers [60]. Since the two layers must have the selfsame areas in a flat bilayer structure, the different layers will be compressed or stretched to achieve suboptimal layer thicknesses, which will result in the internal stress of the asymmetric membrane structure. Therefore, the interfacial tension reaches a maximum value at the interfaces between the two layers. When the surface tension value is close to 0, it follows that except for the junctions of several particles, the other positions are basically in a pressure-free state. For the asymmetric membrane under the strong shear flow, Figure 9b demonstrates that surface tension’s value begins to rise and reaches a stable stage ($\langle \sigma_{z}\rangle =0.0$) between *z* = 10.0$r_{c}$ and *z* = 20.0$r_{c}$, and then decreases to the initial value, indicating that there is almost no pressure inside asymmetric membrane under the strong shear flow. This result is also consistent with the previous experimental results of asymmetric membranes‘ pressures [70]. Differential stresses are caused by suboptimal layer packing densities [60]. In the asymmetric double-layer structure, if the quantity and type of lipids are balanced appropriately between two layers, the intrinsic stress also can be generated. However, under the strong shear flow, the spontaneous curvature of membrane structure is relatively large, the suboptimal packing density of the layer can not reach the state of equilibrium, so its intrinsic stress is almost zero. The resultant force on the membrane interface molecules is not equal to zero, and its resultant force direction is perpendicular to the interior of the membrane structure. As a result, the surface of the membrane tends to shrink automatically, so there is still a certain tension at the interfaces between the solution and membrane. This result confirms the previous work in understanding the non-directional transport of asymmetric membranes in the zero flows [29].

## 4. Summary

In this work, we analyze the shear-induced assembly of asymmetric lipid membranes by a DPD simulation in aqueous solutions based on the CG model under the weak and strong shear flows. Some tubes and vesicles are induced into membranes with $\dot{\gamma }$ = 0.073, while with $\dot{\gamma }$ = 0.168, only membranes and tubes exist in the phase diagram. Then, we investigate the dynamic process of the asymmetric lipid membranes and learn that when reaching the equilibrium state, the asymmetric membrane is more smooth with $\dot{\gamma }$ = 0.073, while the asymmetric membranes will form a certain degree of bending along the shear direction with $\dot{\gamma }$ = 0.168. We consider the average radius of gyration to understand the microstructures’ overall changes and analyze the shape factor to comprehend individual lipid molecules’ changes prominently. By comparing the variation of $\langle R_{g}\rangle$ under the strong and weak shear flows, the oscillation amplitude of the curve, especially of the $R_{\mathrm{gxx}}$ and $R_{\mathrm{gyy}}$ under the strong shear flow is larger than that under the weak shear flow; different shear strengths will affect the shape of the second type of lipid molecules distinctly, while the shape of the first type of lipid molecules is more stable than that of the second type. Additionally, we also analyze the mechanical properties of the asymmetric membrane in solutions by analyzing the interfacial tension. The spontaneous curvature of membrane structure is relatively large with $\dot{\gamma }$ = 0.168, the suboptimal packing density of the layer can not reach the state of equilibrium, so its intrinsic stress is almost zero. The resultant force on the membrane interface molecules is not equal to zero, and its resultant force direction is perpendicular to the interior of the membrane structure. We expect an abundant phase space to consider more system parameters to predict further accurate and luxuriant phase behavior in the future, where the other effects of parameters on the phase behavior should be explored in a multi-dimensional phase diagram.

## Supplementary Materials

Supplementary materials are available online at https://www.mdpi.com/article/10.3390/membranes11090655/s1.
