# Supplementary material for: Asymmetric Lipid Membranes under Shear Flows: A Dissipative Particle Dynamics Study
Source: Membranes (Basel). 2021 Aug 26;11(9):655. doi: 10.3390/membranes11090655 (PMC8465239; doi:10.3390/membranes11090655)
Supplement: Supplementary file 1 [file membranes-11-00655-s001.zip › Supplementary_materials/template.pdf]

Supplementary Materials: Asymmetric Lipid Membranes under Shear Flows: A Dissipative Particle Dynamics Study

Yanying Chen[1], Zhenguo Wang[1], Yongyun Ji[1], Linli He[1], Xianghong Wang[1] and Shiben Li [1,*]

1. The equilibrium state

To ensure that eventually reach the equilibrium state, we have measured the energy $E_{\mathrm{Tot}}/k_{\mathrm{B}}T$, temperature k_{B}, and pressure P inside the simulated box. As shown in Fig. S1, after a trend of decline at first, the energy, pressure, and temperature are basically unchanged during the simulation. Therefore, we can determine that we get the necessary equilibrium state.

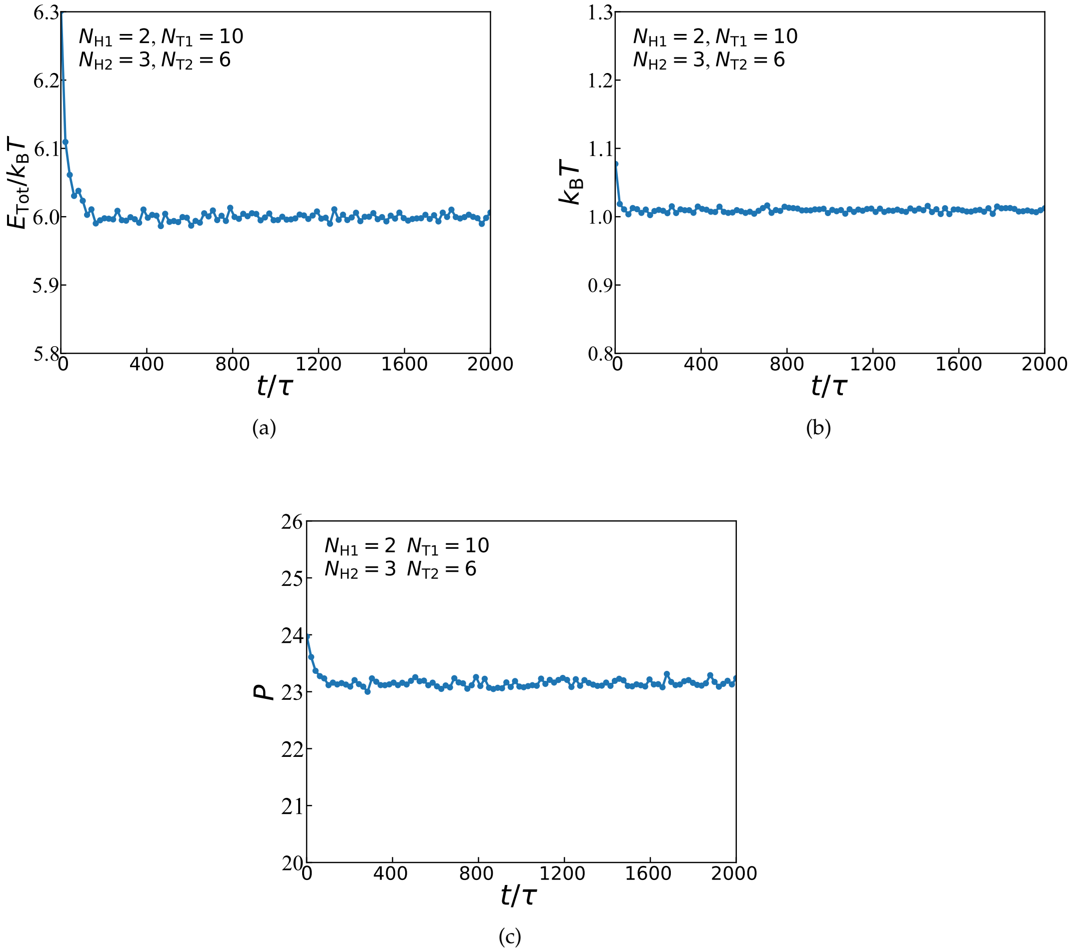

Figure S1. The energy (a), temperature (b) and pressure (c) as functions of time steps for the microstructure.

2. The shear flow

In this simulation, we chose the reverse non-equilibrium molecular dynamics (RNEMD) method, in which the shear rate is the gradient of the fluid velocity along a particular direction. As shown in Fig. S2, when we apply the shear flow along the x-direction, its maximum velocity appears at $z = 0$ and $z = L$, while its minimum velocity appears at $z = L/2$. The periodic simulation box with the length L is subdivided into several slabs in the z-direction, where the atoms inside the slab at $z = 0$ and $z = L/2$ are propelled in two opposite z-directions, respectively. By finding the atom with the highest movement against the desired slab movement, the momentums of the two atoms are interchanged. If the two atoms have the same mass, the non-physical momentum exchange preserves the linear momentum and kinetic energy of the system as a whole. The potential energy and the total energy of the system are conserved because the positions of the atoms are constant. Compared with other methods, there is no energy being deposited into the simulation, hence, it is unnecessary to use an external thermostat to eliminate energy [1].

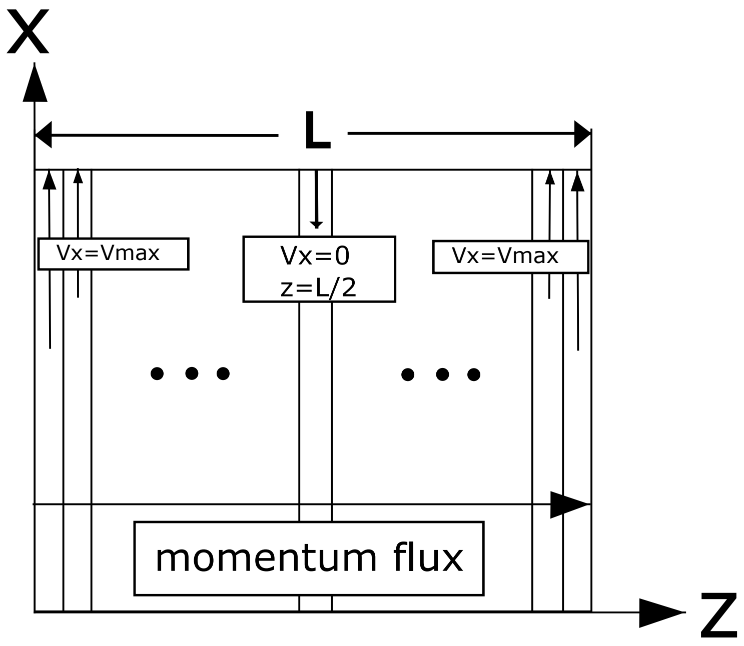

Figure S2. Schematic view of shear flow in the simulation box.

3. The method of density calculation

Along the *z*-axis, we divide the simulation box into 100 layers on average, count the number of each particle in each layer, and divide the result with the volume fraction of each layer to obtain the density distribution curve. To reduce the error, we select 30 kinds of structure data of the equilibrium state that have been reached and calculate their density distribution with the same method. Finally, the average density value of these 30 kinds of data is obtained, and the density distribution curve is drawn.

4. The gyration tensor

To more accurately quantify the shape, we calculate the three-dimensional gyration tensor of the polymer, which is defined as follows [2]:

$$\mathbf{R}_g^2 = \begin{bmatrix} R_{gxx}^2 & R_{gxy}^2 & R_{gxz}^2 \\ R_{gyx}^2 & R_{gyy}^2 & R_{gyz}^2 \\ R_{gzx}^2 & R_{gzy}^2 & R_{gzz}^2 \end{bmatrix} \rightarrow \begin{bmatrix} L_1^2 & 0 & 0 \\ 0 & L_2^2 & 0 \\ 0 & 0 & L_3^2 \end{bmatrix} \quad (1)$$

where the element of $R_{g\alpha\beta}^2$ follows as:

$$\left\langle R_{g\alpha\beta}^2 \right\rangle = \frac{1}{N} \sum_i \left\langle \left(r_{i,\alpha} - r_{c,\alpha}\right)\left(r_{i,\beta} - r_{c,\beta}\right) \right\rangle \quad (2)$$

Based on the gyration tensor, we can obtain the gyration radius of the polymer, which can be calculated as follows:

$$R_g^2 = \frac{1}{N} \sum_{i=1}^{N} (\mathbf{r}_i - \mathbf{r}_c)^2 = L_1^2 + L_2^2 + L_3^2 \quad (3)$$

In order to clearly show the effect of shear flow on the microstructure, three components of the average radius of gyration are calculated and the anisotropic conformations of lipid molecules under strong and weak shear are compared.

5. The shape factor

Imagining that a unit mass is located at every one of the N steps of the walk, the tensor $\overline{\boldsymbol{T}}$ with components can be constructed as:

$$T_{ij} = \frac{1}{N}\sum_{l=1}^{N}(\bar{X}_{il} - \bar{X}_i)\left(X_{jl} - \bar{X}_j\right) \quad = \frac{1}{N^2}\sum_{l>R}^{N}(X_{il} - X_{ik})\left(X_{jl} - X_{jk}\right) \tag{4}$$

For three-dimensional walks, the matrix has three eigenvalues, L_1^2, L_2^2, L_3^2, the three eigenvalues of $\mathbf{R}_g^2$. The matrix also has three invariants, T_r, M and D, where T_r and D are the trace and determinant, respectively, and M is the sum of its three minors:

$$\begin{aligned} \mathrm{Tr} &= L_1^2 + L_2^2 + L_3^2 \\ \mathrm{D} &= L_1^2 L_2^2 L_3^2 \\ \mathrm{M} &= L_1^2 L_2^2 + L_1^2 L_3^2 + L_2^2 L_3^2 \end{aligned} \tag{5}$$

Then we define the shape factor expression as:

$$\langle\delta\rangle = \frac{\left\langle\left(\mathrm{Tr}^2 - 3\mathrm{M}\right)\right\rangle}{\left\langle\mathrm{Tr}^2\right\rangle} = 1 - 3\frac{\langle L_1^2 L_2^2 + L_2^2 L_3^2 + L_3^2 L_1^2\rangle}{\left\langle\left(L_1^2 + L_2^2 + L_3^2\right)^2\right\rangle} \tag{6}$$

As for the shape factors, an alternative definition makes sense:

$$\langle\delta\rangle = 1 - 3\left\langle\frac{L_1^2 L_2^2 + L_2^2 L_3^2 + L_1^2 L_3^2}{\left(L_1^2 + L_2^2 + L_3^2\right)^2}\right\rangle \tag{7}$$

(See [3,4] for the detailed derivation process, which is briefly summarized here.)

6. Tension

The instantaneous pressure tensor is defined as:

$$p_{\beta\alpha} = p^{\text{kin}}_{\beta\alpha} + p^{\text{int}}_{\beta\alpha} \tag{8}$$

where $p^{\text{kin}}_{\beta\alpha}$ represent a kinetic contribution from throughput of linear momentum resulting from the particle velocities and $p^{\text{int}}_{\beta\alpha}$ represent an internal contribution from intermolecular and intramolecular forces. The pressure due to internal forces is given as:

$$\bar{p}^{\text{int}}_{\beta\alpha}(j) = \frac{1}{A_{\alpha j}}\left(F_{j\beta} + F_{j+1\beta} + \cdots + F_{N\beta}\right) \tag{9}$$

when $\alpha_0 = \sum_{i=1}^{N+1} \Delta\alpha_i$, then:

$$\bar{p}^{\text{int}}_{\beta\alpha} = \frac{1}{\alpha_0}\sum_{i=1}^{N+1} \bar{p}^{\text{int}}_{\beta\alpha}(i) \cdot \Delta\alpha_i = \frac{1}{A_\alpha}\frac{1}{\alpha_0}\sum_{i=1}^{N}\left(F_{i\beta} + F_{i+1\beta} + \cdots + F_{N\beta}\right)\Delta\alpha_i \tag{10}$$

Now we rearrange this expression from a sum over all N planes into a sum over all N particles and $\alpha_i = \Delta\alpha_1 + \cdots + \Delta\alpha_i$, $V = A_\alpha\alpha_0$:

$$\bar{p}^{\text{int}}_{\beta\alpha} = \frac{1}{V}\sum_{i=1}^{N} F_{i\beta}\alpha_i \tag{11}$$

The kinetic contribution of the pressure is defined as:

$$\bar{p}^{\text{kin}}_{\beta\alpha}(\alpha) = \frac{1}{A_\alpha\Delta\alpha}\left\langle \sum_{\alpha-\Delta\alpha/2\leqslant\alpha_i\leqslant\alpha+\Delta\alpha/2} m_i v_{i\beta} v_{i\alpha} \right\rangle \tag{12}$$

Then we average over all cross sections of an equal small width $\Delta\alpha$ from $\alpha = 0$ to $\alpha = \alpha_0$ to obtain:

$$\bar{p}^{\text{kin}}_{\beta\alpha} = \frac{1}{V}\sum_{i=1}^{N} m_i v_{i\beta} v_{i\alpha} \tag{13}$$

Hence we can get the complete result:

$$\bar{p}_{\beta\alpha} = \frac{1}{V}\left(\sum_{i=1}^{N} m_i v_{i\beta} v_{i\alpha} + \sum_{i=1}^{N} F_{i\beta}\alpha_i\right) \tag{14}$$

(See [5] for the detailed derivation process, which is briefly summarized here.)

1. Müller-Plathe, F. Reversing the perturbation in nonequilibrium molecular dynamics: An easy way to calculate the shear viscosity of fluids. *Physical Review E* **1999**, *59*, 4894–4898
2. Huang, W.; Zaburdaev, V. The shape of pinned forced polymer loops. *Soft Matter* **2019**, *15*, 1785–1792
3. Rudnick, J.; Gaspari, G. The asphericity of random walks. *Journal of Physics A: Mathematical and General* **1986**, *19*, L191–L193
4. Zifferer, G.; Preusser, W. Monte Carlo Simulation Studies of the Size and Shape of Ring Polymers. *Macromolecular Theory and Simulations* **2001**, *10*, 397–407
5. Heinz, H.; Paul, W.; Binder, K. Calculation of local pressure tensors in systems with many-body interactions. *Physical Review E* **2005**, *72*, 066704
