# Supplementary figures and images for: Asymmetric Lipid Membranes under Shear Flows: A Dissipative Particle Dynamics Study
Source: Membranes (Basel). 2021 Aug 26;11(9):655. doi: 10.3390/membranes11090655 (PMC8465239; doi:10.3390/membranes11090655)

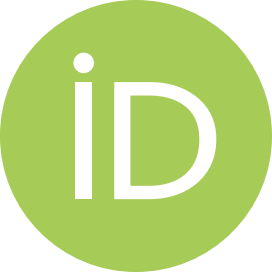

Supplement: Supplementary file 1 [file membranes-11-00655-s001.zip › Supplementary_materials/Definitions/logo-orcid-eps-converted-to.pdf]

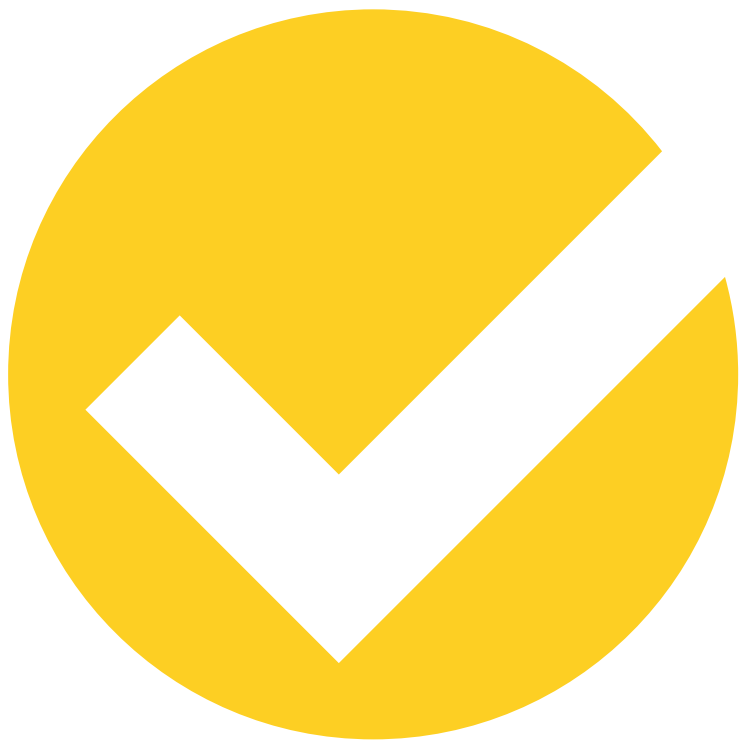

check for  
updates

Supplement: Supplementary file 1 [file membranes-11-00655-s001.zip › Supplementary_materials/Definitions/logo-updates.pdf]

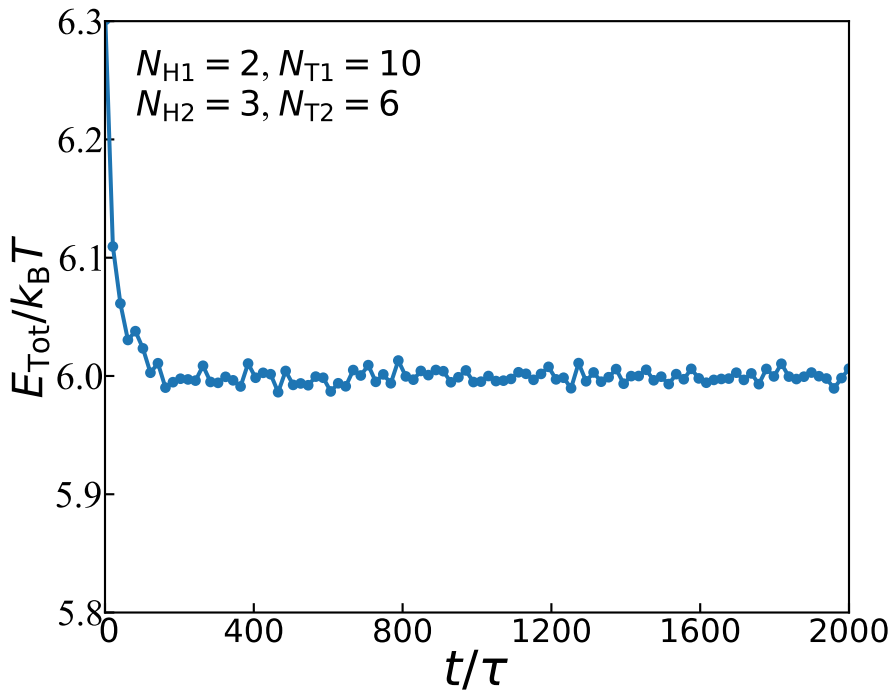

Supplement: Supplementary file 1 [file membranes-11-00655-s001.zip › Supplementary_materials/E-eps-converted-to.pdf]

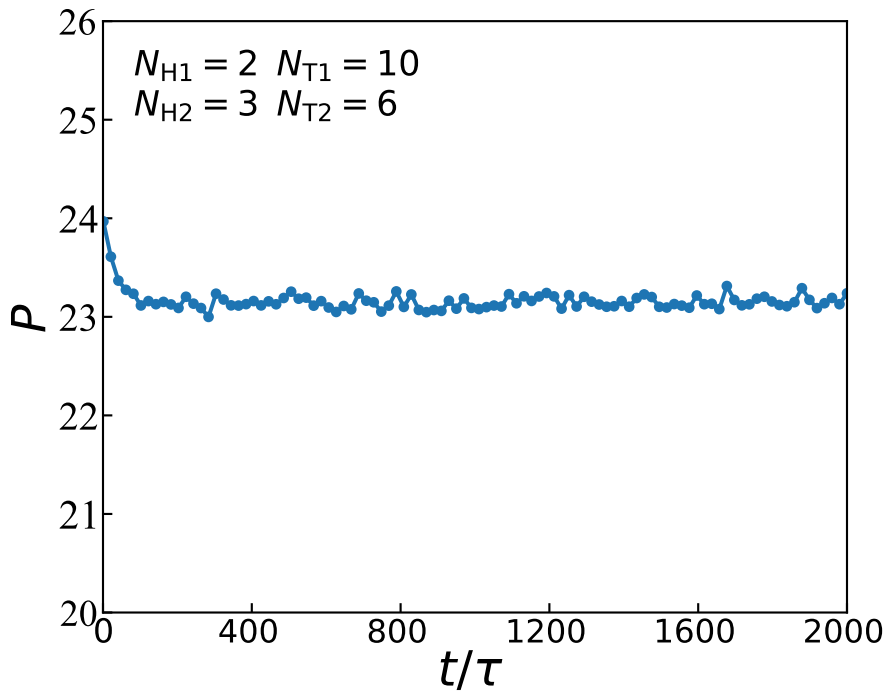

Supplement: Supplementary file 1 [file membranes-11-00655-s001.zip › Supplementary_materials/P-eps-converted-to.pdf]

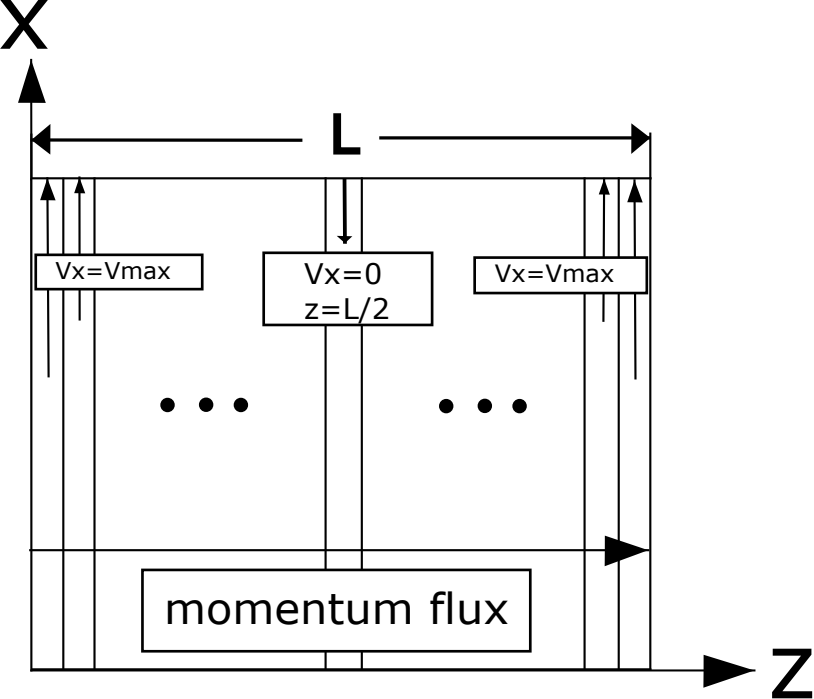

Supplement: Supplementary file 1 [file membranes-11-00655-s001.zip › Supplementary_materials/shear_view-eps-converted-to.pdf]

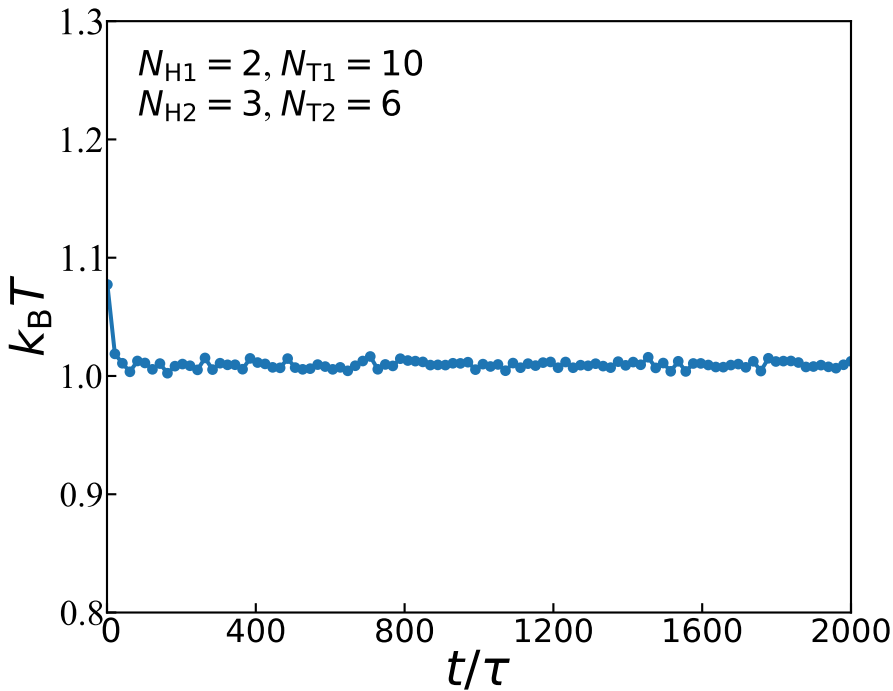

Supplement: Supplementary file 1 [file membranes-11-00655-s001.zip › Supplementary_materials/T-eps-converted-to.pdf]
